# Prospective study on surgical site infections in major oncologic resections

**DOI:** 10.6026/973206300213231

**Published:** 2025-09-30

**Authors:** Poornima Venkatraman, Vishal Mruthyunjaya Kalmani, Arundhati Maindola, Yeruva Bheemeswara Reddy, Dhyaneshwar Ra, Malavika Sajeev

**Affiliations:** 1 Department of Surgery, Davao Medical School Foundation, Philippines, Telangana, India; 2 Department of Emergency Medicine, Barking Havering and Redbridge University Hospitals NHS Trust, Essex, United Kingdom; 3 Department of Health and Family Welfare, Directorate of Health Safety and Regulations, Shimla, Himachal Pradesh, India; 4 Department of Surgery, Santhiram Medical College, Andhra Pradesh, India; 5 Department of General Surgery, Government Stanley Medical College Chennai, Tamil Nadu, India; 6 Department of Emergency Medicine, East Sussex NHS Healthcare Trust, Eastbourne, United Kingdom

**Keywords:** Surgical site infection, oncology surgery, risk factors, prospective study, microbiological profile, infection prevention, cancer resection

## Abstract

The incidence, risk factors, and microbiological profile of surgical site infections (SSIs) in 142 patients undergoing major oncologic resections across gastrointestinal, breast, and head and neck malignancies is of interest. The overall SSI rate was 19.7%, with the highest incidence seen in gastrointestinal surgeries. Prolonged operative time, high ASA grade, and preoperative hypoalbuminemia were significant predictors of SSI. Most infections were caused by gram-negative organisms, with *E. coli* and *Klebsiella* species predominant. Thus, the importance of preoperative optimization and tailored antibiotic strategies in oncology surgery is shown.

## Background:

Surgical site infections (SSIs) are among the most common postoperative complications, particularly in patients undergoing major oncologic resections [1]. These infections not only increase patient morbidity and hospital stay but also delay adjuvant therapy, ultimately impacting long-term oncologic outcomes [2]. Despite advances in surgical techniques and perioperative care, SSI rates in cancer patients remain significantly higher compared to non-oncologic surgical populations [3]. This elevated risk is multifactorial, often attributed to factors such as immunosuppression from malignancy, nutritional deficits, chemotherapy effects, and complex, prolonged surgeries [4]. Oncologic surgeries typically involve extensive resections with or without reconstruction, frequently resulting in prolonged operative times, greater tissue handling, and potential for contamination-especially in gastrointestinal and head & neck procedures [5]. The microbial spectrum is also influenced by anatomical site and hospital flora, necessitating precise local data to guide effective prophylactic and therapeutic strategies [6]. Although numerous retrospective studies have addressed SSI risk, prospective data focused specifically on the oncology surgical population is limited [7]. Therefore, it is of interest to prospectively evaluate the incidence, contributing factors, and microbiological patterns of surgical site infections following major cancer surgeries, with the ultimate goal of identifying modifiable risks and informing targeted interventions to reduce postoperative infectious morbidity in this vulnerable population.

## Materials and Methods:

This prospective observational study was conducted over a 20-month period from March 2023 to October 2024, in the surgical oncology department of a tertiary cancer center. A total of 142 patients undergoing elective major oncologic resections were enrolled consecutively after obtaining informed consent. Inclusion criteria included patients aged ≥18 years undergoing definitive surgical resection for malignancies of the gastrointestinal tract, breast, or head and neck region. Patients with pre-existing infections, emergency surgeries, palliative procedures, or those receiving long-term immunosuppressive therapy were excluded. All surgeries were performed by experienced oncologic surgeons using standardized aseptic techniques. Preoperative variables such as age, sex, body mass index (BMI), ASA grade, nutritional status (including serum albumin), comorbidities (diabetes mellitus, anemia), neoadjuvant therapy, and smoking status were documented. Operative parameters included surgical site, type of surgery (clean, clean-contaminated, contaminated), use of drains, blood loss, operative duration, and perioperative antibiotic prophylaxis. SSIs were classified according to CDC guidelines into superficial incisional, deep incisional, and organ/space infections. Patients were followed for 30 days postoperatively with daily wound inspection during hospitalization and subsequent outpatient review. When infection was suspected, wound swabs or pus cultures were obtained and analyzed using standard microbiological techniques. Antibiotic sensitivity testing was performed for all isolates. The primary outcome was the incidence of SSI. Secondary outcomes included identification of independent risk factors and characterization of the microbiological spectrum of infections. Data were analyzed using SPSS version 26. Univariate and multivariate logistic regression were applied to identify predictors of SSI, with p<0.05 considered statistically significant.

## Results:

A total of 142 patients undergoing major oncologic resections were included in this prospective study. The overall incidence of surgical site infections (SSIs) was 19.7% (n = 28). SSI rates were highest among gastrointestinal surgeries (28.3%), followed by head and neck (17.6%) and breast surgeries (8.1%). Multivariate analysis revealed that prolonged operative duration, ASA grade ≥3, and preoperative hypoalbuminemia (<3.5 g/dL) were independent predictors of SSI. Most infections were superficial, and the predominant organisms isolated were *Escherichia coli*, *Klebsiella pneumoniae*, and *Staphylococcus aureus*. Table 1 (see PDF) summarizes the baseline demographic and clinical characteristics of the 142 enrolled patients. The mean age was 56.2 years, with a slight male predominance. Nearly one-third had ASA grade ≥3, 33.1% had hypoalbuminemia, and 43% had preoperative anemia-factors associated with increased infection risk. Table 2 (see PDF) presents the distribution of surgeries by cancer type and their corresponding SSI rates. Gastrointestinal resections accounted for the largest share and also had the highest SSI incidence at 28.3%. Head and neck and sarcoma surgeries followed, while breast cancer procedures had the lowest infection rate (8.1%). Table 3 (see PDF) classifies SSIs by type, showing that superficial infections were most common (50%), followed by deep incisional (28.6%) and organ/space infections (21.4%), indicating that the majority of infections were manageable with local wound care. Table 4 (see PDF) compares risk variables between the SSI and non-SSI groups. Patients who developed SSIs had significantly longer operative times, higher rates of hypoalbuminemia (64.3% vs. 25.4%), and were more likely to have ASA grade ≥3, all with statistically significant p-values. Table 5 (see PDF) reports SSI incidence by wound classification. Clean-contaminated and contaminated surgeries had substantially higher infection rates (27.8% and 29.6%, respectively) compared to clean procedures (8.2%), emphasizing the role of intraoperative contamination in SSI pathogenesis. Table 6 (see PDF) presents a multivariate logistic regression model identifying three independent predictors of SSI: operative time >180 minutes, ASA grade ≥3, and preoperative hypoalbuminemia. Each was associated with significantly increased odds of infection. Table 7 (see PDF) describes the microbiological profile of SSI cases. Gram-negative organisms were predominant, with *Escherichia coli* (32.1%) and *Klebsiella pneumoniae* (25%) leading, followed by *Staphylococcus aureus* (21.4%). Polymicrobial infections occurred in 10.7% of cases. Table 8 (see PDF) outlines antibiotic resistance patterns. The majority of *E. coli* and Klebsiella isolates were resistant to ceftriaxone (77.8% and 85.7%, respectively), highlighting growing resistance among gram-negative organisms. Carbapenem resistance remained relatively low. Table 9 (see PDF) shows that patients with SSIs had a significantly longer hospital stay, with a median duration of 13 days versus 7 days in non-infected patients, contributing to increased healthcare burden and resource utilization. Table 10 (see PDF) compares postoperative antibiotic use. The mean duration of antibiotics was significantly higher in patients with SSIs (9.3 days) compared to those without (3.8 days), indicating increased treatment intensity and potential risk of antimicrobial resistance.

## Discussion:

This prospective study highlights a substantial burden of surgical site infections (SSIs) in patients undergoing major oncologic resections, with an overall incidence of 19.7%. Notably, gastrointestinal surgeries exhibited the highest SSI rate (28.3%), aligning with existing literature attributing elevated infection risk to the involvement of enteric contents and prolonged operative times. Breast surgeries, typically clean procedures, demonstrated the lowest infection rate (8.1%), affirming the role of wound classification in SSI risk stratification [8]. The identification of hypoalbuminemia, elevated ASA grade, and operative time >180 minutes as independent predictors underscores the multifactorial nature of SSIs [9]. Particularly for cancer patients who are prone to cachexia, this study supports preoperative nutritional management. Another important impact was the length of the surgery. Extended procedures frequently indicate more intricate surgery and more tissue manipulation both of which increase the risk of microbial contamination and impede wound healing. Similarly, markedly greater infection prevalence was linked to an ASA grade of ≥3, which indicates a larger systemic illness burden [10].

Individualized risk reduction methods and perioperative planning should be informed by these factors. Gram-negative organisms predominated, according to microbiological profile, with *Staphylococcus aureus* coming in second, followed by *Escherichia coli* and *Klebsiella pneumoniae*. Concerns regarding empirical antibiotic selection were raised by the startling discovery that a large number of gram-negative isolates were resistant to third-generation cephalosporins [11]. In high-risk surgical oncology cases, these resistance patterns point to the need to reevaluate local antibiotic prophylactic strategies in favor of medicines that are effective against resistant gram-negatives. The implications were clinically severe even though the infection rate was mild. SSI patients needed greater antibiotic durations and had a median hospital stay that was almost twice as long as that of non-infected patients [12]. Such results may jeopardize long-term oncologic outcomes by delaying surgical recovery and the start of adjuvant therapy [13]. Our study confirms that SSIs continue to be a major concern in oncologic surgery, particularly for patients who are nutritionally fragile and undergo gastrointestinal resections. Infection rates can be significantly decreased by interventions such preoperative nutritional correction; careful surgical planning and tailored antibiotic tactics based on local resistance data. Future studies should examine the role of cutting-edge preventative techniques in high-risk patients, such as antimicrobial sutures and negative pressure wound care.

## Conclusion:

Surgical site infections remain a significant concern following major oncologic procedures, particularly in gastrointestinal cancer surgeries. Addressing modifiable risk factors such as hypoalbuminemia and prolonged operative time, alongside optimizing antibiotic prophylaxis, can substantially improve postoperative outcomes in cancer patients.
